# Apoptosis characterization in mononuclear blood leukocytes of HIV patients during dengue acute disease

**DOI:** 10.1038/s41598-020-62776-4

**Published:** 2020-04-14

**Authors:** Amanda Torrentes-Carvalho, Juan Camilo Sánchez-Arcila, Tamiris Azamor, Luciana Santos Barbosa, Eugênio Damaceno Hottz, Mariana Gandini, Fernando Augusto Bozza, Rivaldo Venâncio da Cunha, Luzia Maria de Oliveira Pinto, Paulo Vieira Damasco, Elzinandes Leal de Azeredo

**Affiliations:** 1 Departamento de Imunobiologia, Instituto de Biologia -Universidade Federal Fluminense (UFF), Niterói, Brazil; 2 Laboratório de Imunologia Viral, Instituto Oswaldo Cruz (IOC) – Fundação Oswaldo Cruz (FIOCRUZ), Rio de Janeiro, Brazil; 3 Laboratório de Tecnologia Imunológica - Fundação Oswaldo Cruz (FIOCRUZ), Rio de Janeiro, Brazil; 4 Laboratório de Imunofarmacologia, Instituto Oswaldo Cruz (IOC) – Fundação Oswaldo Cruz (FIOCRUZ), Rio de Janeiro, Brazil; 5 Laboratório de Microbiologia Celular, Instituto Oswaldo Cruz (IOC) – Fundação Oswaldo Cruz (FIOCRUZ), Rio de Janeiro, Brazil; 6 Laboratório de Medicina Intensiva, Intituto Nacional de Infectologia Evandro Chagas-INI, Fundação Oswaldo Cruz (FIOCRUZ), Rio de Janeiro, Brazil; 7 Universidade Federal do Mato Grosso do Sul (UFMS), Faculdade de Medicina, Campo Grande, Mato Grosso do Sul Brazil; 8 Hospital Universitário Gafreé & Guinle, Universidade Federal do Estado do Rio de Janeiro (UNIRIO), Rio de Janeiro, Brazil; Hospital Pedro Ernesto, Universidade Estadual do Rio de Janeiro (UERJ), Rio de Janeiro, Brazil

**Keywords:** Immunology, Immunology, Infectious diseases, Infectious diseases

## Abstract

Dengue virus (DENV) co-circulation in Brazil represents a challenge for treatment and vaccine development. Despite public health impact, the occurrence of coinfections with other viruses is a common event. Increased T cell activation and altered inflammatory response are found during DENV coinfection with Human Immunodeficiency Virus (HIV) impacting HIV-pathogenesis. Even with Antiretroviral therapy (ART), HIV- treated patients had chronic immune activation and lymphocyte apoptosis. However, apoptotic mechanisms have not been investigated during coinfection with DENV. Our attention was attracted to apoptotic cell markers expressions in PBMCs from DENV and DENV/HIV coinfected patients. We found CD4/CD8 ratio inversion in most coinfected patients. CD4 T and CD8 T-cell subsets from DENV and DENV/HIV groups expressed low levels of anti-apoptotic protein Bcl-2. Furthermore, CD8 CD95 double positive cells frequency expressing low levels of Bcl-2 were significantly higher in these patients. Additionally, the density of Bcl-2 on classical monocytes (CD14^++^CD16^−^) was significantly lower during DENV infection. Upregulation of pro-apoptotic proteins and anti-apoptotic proteins were found in DENV and DENV/HIV, while catalase, an antioxidant protein, was upregulated mainly in DENV/HIV coinfection. These findings provide evidence of apoptosis triggering during DENV/HIV coinfection, which may contribute to knowledge of immunological response during DENV acute infection in HIV-patients treated with ART.

## Introduction

Dengue virus (DENV) and Human Immunodeficiency Virus (HIV) infections are currently a major public health concern in Brazil. Dengue is caused by any one of four DENV (1–4) serotypes spread by mosquitoes of *aedes* genus. DENV causes an acute febrile illness of a broad clinical spectrum presenting both asymptomatic and symptomatic forms that can evolve with severe and potentially fatal condition^1^. The hallmark of most serious clinical conditions is the vascular permeability increase that causes plasma leakage, leading to shock and death. Acquired Immunodeficiency Sydrome (AIDS) (is still considered an important cause of morbidity and mortality in the world. HIV infects CD4 T-cells, monocytes/macrophages and to a lesser extent in dendritic cells leading to progressive CD4 T-cell depletion and consequently pronounced immunosuppression in the absence of effective antiretroviral therapy (ART)^2^.

Apoptosis, also called type I cell death, is a regulated  process of cell death highly conserved among mammals and comprises a controlled self-destruction process to eliminate damaged, neoplastic and virus-infected cells^3^. Apoptosis program regulation is determined by interactions of three members of B-cell lymphoma-2 family proteins (Bcl-2): Bcl-2 homologous 3 (BH3)-only proteins, Bcl-2 proteins and Bcl-2 associated X protein (Bax)/Bcl-2 homologous antagonist/killer (Bak) effectors. It can be triggered by extrinsic [death receptors like p55 tumor necrosis factor TNF receptor- TNFRI, Fas/CD95, TRAIL/APO2-L (TNF-related apoptosis-inducing ligand) receptors 1 and 2] and intrinsic pathways (mitochondrial membrane associated proteins and released factors)^4^. Regardless of how it is initiated, both pathways result in caspases activation that culminate in cellular disruption^5^.

Apoptosis deregulation is an important factor for CD4 T- cells depletion and disease pathogenesis during HIV^6^. In relation to dengue infection, lymphopenia is frequently reported in infected patients^7,8^ and impaired cellular activation, migration^9^ and apoptosis^10^ have been considered a likely explanation of it. Previously, it has been proposed by our group and others that apoptosis is triggered by DENV replication^11^. In this context, we have shown that *in vitro* infection of human monocytes by DENV-2 triggers apoptotic cell death phenotype^12^. In addition, T lymphocyte apoptotic markers up-regulation was associated with cell activation in naturally infected DENV patients^13^. Importantly, coinfected patients with DENV and HIV presented increased CCR5 and CD107a expressions on T –cell subsets indicating T cell activation/cytotoxicity and migration^14^. However, cell phenotype associated with apoptosis was not evaluated before.

Considering that apoptosis may be involved in the DENV and HIV immunopathology, we assessed the frequency of apoptotic cell markers in circulating mononuclear leukocytes comparing groups of DENV, HIV monoinfected and DENV/HIV coinfected patients. Our data demonstrate that most treated HIV patients diagnosed with acute dengue disease had lower CD4 absolute counts and inverted CD4/CD8 T- cell ratio. With regard to apoptotic cell markers, CD4 T and CD8 T-cell subsets from DENV and DENV/HIV groups expressed low levels of anti-apoptotic molecule Bcl-2. Importantly, death receptor Fas/CD95 was up-regulated mainly on T- cells expressing low levels of Bcl-2. Monocyte subsets analysis showed decreased CD14^++^ CD16^−^ classical monocytes frequency during DENV infection and increased CD14^++^ CD16^+^ intermediate ones in monoinfection and coinfection as well. The density of Bcl-2 on classical monocytes from DENV infected patients was lower as compared to coinfected ones. As demonstrated by apoptosis-related protein expression screening analysis, peripheral blood mononuclear cells (PBMCs) from DENV patients had increased expression of pro- and anti-apoptotic molecules. Additionally, DENV and DENV/HIV groups had increased expression of Bad and Bax pro-apoptotic proteins whereas catalase antioxidant protein was upregulated mainly in DENV/HIV. Our data suggest that immune scenario generated as a result of coinfection with HIV may be interfering in cell activation and death susceptibility during acute dengue infection. Further studies evaluating immune response of ART treated coinfected patients are of great importance for prevention and treatment of both infections.

## Materials and methods

### Study population

The study was carried out during DENV-1, DENV-2 and DENV-4 outbreaks in Brazil. Patients were enrolled in this study between 2011–2013 upon admission to different hospitals in the city of Rio de Janeiro as already described^14^. During the study period, 43 cases of Dengue were included and submitted to investigation. Participants were grouped according to HIV and DENV status as follows: DENV monoinfection (n = 20) and DENV/HIV coinfection (n = 23). Ten (10) HIV positive individuals were include and considered as HIV monoinfection. All HIV infected patients were receiving ART therapy. Ten (10) healthy donors (HD) were enrolled in the study as controls. Suspected cases of dengue infection were considered when patients presented acute febrile illness, with a maximum duration of 7 days, accompanied by at least 2 of the signs or symptoms such as headache, retro- orbital pain, myalgia, arthralgia, prostration or rash, associated or not with the presence of bleeding, with an epidemiological history for dengue infection^1^. Dengue confirmed cases were classified according to the latter WHO guideline in the following groups: Dengue without warning signs (DwoWS), Dengue with warning signs (DwWS) and Severe dengue (SD)^1^.

### Laboratorial diagnosis

The diagnosis of dengue infection was confirmed by enzyme-linked immunosorbent assay ELISA-IgM (Panbio, inc, USA); detection of non-structural 1 protein (NS1) protein by Platelia Dengue NS1 antigen. Molecular detection and serotype typing were performed by conventional RT-PCR as described previously^15^. The immune response to Dengue was considered as primary or secondary by IgG ELISA (Focus Diagnosis, Cypress, CA, USA). The HIV diagnosis was confirmed by Abbott Real Time HIV-1 Qualitative amplification assay.

The National Commission for Research Ethics in Brazil (Plataforma Brasil- CAAE 57221416.0.1001.5248; Instituto de Pesquisas Clinicas Evandro Chagas, Fiocruz-CAAE 3723.0.000.009-08 and Hospital Universitário Pedro Ernesto -2144-CEP/HUPE) have already approved the recruitment of patients to carry out the proposed study. Experiments were performed in compliance with those protocols. Written informed consent was obtained from participants and/or legal guardians prior to any procedure in accordance with the National Commission for Research Ethics.

### Human peripheral blood mononuclear cells (PBMCs) purification and cryopreservation

PBMCs were isolated and purified by density gradient centrifugation (Ficoll–Paque; Sigma), resuspended in freezing solution (Fetal bovine serum - FBS containing 10% dimethyl sulfoxide) and cryo-preserved in liquid nitrogen. The plasma was aliquoted and stored at −70 °C.

### Peripheral blood mononuclear cells phenotypic assessments

Eight-color flow cytometry method was performed using the following monoclonal antibodies: anti-CD4APC Cy7 (BioLegend); anti-CD8BV650 (BD Biociences); anti-CD3Alexa Fluor 700 (eBiocience); anti-CD95FITC (Southern Biotech); anti-Bcl-2PE (BD Biociences), anti-CD14- BV 500 (eBiocience), CD16 PE Cy7 (BioLegend). Far Red Dead cell stain was applied to exclude dead cells (Invitrogen). A matching isotype control for each antibody was included in all experiments.

### Extracellular and intracellular labelling for flow cytometry analysis

Cryopreserved PBMCs (0.5–106) from patients and controls were thawed in water bath at 37 °C, centrifuged (350 g, 5 min) and washed once with 1 mL of PBS pH 7.4 supplemented with 2% FCS and 0,01% NaN3. Thawed cells were stained with panels comprising the antibodies listed above for flow cytometry analysis. Cells were labeled extracellularly and intracellularly as described previously^13^. Briefly, after extracellular stain with cocktail surface markers, cells were washed in Perm/Wash for 15 min and then were incubated with Cytofix/Cytoperm solution for 20 min at 4 °C (BD biosciences). Cells were washed again in Perm/Wash and stained for 30 min with PE conjugated anti apoptotic Bcl-2 protein mAb. About 1 × 10^5^ events were acquired in the lymphocyte and monocyte gates. Data acquisition and analysis were performed using FACS Aria IIu and analyzed using FlowJo software version 10 respectively.

### Expression profile assay of proteins with apoptotic and anti-apoptotic functions

Apoptosis–related proteins expression profile was analyzed using Human Apoptosis Array Kit (R&D Systems). PBMCs lysates were obtained from DENV monoinfected, HIV monoinfected and DENV/HIV coinfected, as well as HD. The lysate volume was adjusted for 250 μL/array, according to the manufacturer protocol. Two kits, both containing 4 nitrocellulose membranes each with 35 different anti-apoptosis antibodies printed in duplicate, were performed. After washes and immunodetection antibodies procedures, each membrane was treated with chemiluminescent reagents, covered with plastic wrap and exposed to X-ray film for 5–10 minutes. The positive signals on developed film were identified by placing the transparency overlay on the array image and aligning it with the three pairs of positive control spots in the corners of each array. Apoptosis array data on developed X-ray film were quantified by scanning the film on a transmission-mode scanner and the array image file was evaluated using image analysis software quantity One (Bio-Rad Version 4.6.3). A template analyze pixel density in each spot of the array was created and subtraction of averaged background signal from each one performed. Data were export to Microsoft Excel and the average signal (pixel density) of the duplicate spots pairs, representing each apoptosis-related protein, calculated. Corresponding signals of each duplicate spot were determined and compared to analyses changes in apoptosis-related protein levels between controls and patient samples. Finally, normalization of the signal intensity based on positive and negative controls values were performed.

### Statistical analysis

Statistical analyses were performed by Anova analysis. Kruskal wallis and Dunn’s multiple comparison tests were used for all pairs of groups using GraphPad P sofware (version 6). P values < 0.05 were considered statistically significant. A two-dimensional heat map with hierarchical clustering was built, with the expression of the apoptotic markers in the Y dimension and the HIV, DENV and DENV/HIV groups in the X dimension. The cluster for protein markers was constructed using the Euclidean distance between the medians of the scaled values, and Ward as a linkage algorithm. We ran 1000 bootstrap replications to verify the clustering support. We constructed a Principal Component Analysis (PCA) to verify the distribution pattern of the studied analytes among the individuals. This technique reduces the dimensionality space to reveal the analytes that contribute more to a group profile differentiation. Each Principal Component (PC1 and PC2) represents the total variation (%) explained the distribution of the individuals along with the multidimensional space. In the PCA plot, the individuals are represented as colored points, and the variables are drawn as arrows. The length of each arrow is proportional to the contribution of each variable to the overall variance. In this plot, the angles formed by two arrows (variables) represent the correlation between them and the closer the angle, the higher the correlation between them from orthogonal, independent variables, to more collinear, highly correlated pairs. Cluster and bootstrap analyses were performed using packages gplots^16^ and pvclust^18^ respectively, and PCA analysis was performed using *vegan*^19^, all of them in the statistical environment^17^.

## Results

### Demographic, clinical and laboratorial characteristics

We prospectively included forty-three (43) patients infected with DENV during acute phase of infection [days after disease onset median 5 (1–12) min-max]. Four groups were analyzed: patients monoinfected by DENV (n = 20), coinfected by DENV and HIV (n = 23), patients infected by HIV (n = 10) and HD (n = 10). All HIV infected and coinfected were receiving ART according to Brazilian guidelines and presented undetectable viral load (<50 copies). Nucleoside reverse-transcriptase inhibitors (NRTI), Non-nucleoside reverse-transcriptase inhibitor (NNRTI), Protease inhibitors (PI) and Integrase inhibitors (INI) were ART schemes applied.

There were no significant statistical differences in age, sex or other signs/symptoms between groups of patients analyzed. The Dengue infected and coinfected patients enrolled presented fever accompanied by one or more signs/symptoms such as myalgia, arthralgia, exanthema, headache, prostration, pruritus, conjunctival hyperemia, edema, nausea, vomiting, and retro orbital pain. Dengue monoinfected patients were classified according to the latest WHO classification^1^. Of these, 20 were classified as DwoWS, 17 as DwWS, and 6 as SD. According to groups mono or coinfected, we observed that 6 DENV monoinfected presented DwoWS, 9 patients DwWS and 5 were classified as SD. With respect to DENV/HIV coinfected patients, 14 had DwoWS, 8 DwWS and only one were classified as SD. Regardless mono DENV or DENV/HIV coinfected, the main warning signs presented by infected patients were abdominal pain, mucosal bleeding, liver enlargement and increased hematocrit concomitant with decreased platelets counts. Severe patients presented persistent abdominal pain, followed by uncontrollable vomiting, severe bleeding and severe plasma leakage. Most patients infected with dengue had a positive IgG reaction suggesting a previous heterologous DENV infection. According to laboratorial parameters, DENV monoinfected patients presented low platelet counts compared to HIV (p < 0.01) and high ALT levels compared to DENV/HIV patients (p < 0.05). DENV/HIV coinfected patients presented low leukocytes counts compared to HIV ones (p < 0.05). Demographic, clinical and laboratorial information are summarized in Table 1.

**Table 1:** Demographic, clinical and laboratorial characteristics of DENV, DENV/HIV coinfected and HIV infected patients.

	DENV n = 20	DENV/HIV n = 23	HIV n = 10
Age median (min-max)	39 (19–71)	41 (18–58)	50.5 (23–69)
Days of disease	5 (3–6)	5 (4–6)	—
Gender F: M, n (%)	12 (60%)/8(40%)	9 (39.1%)/14(60.8%)	4 (40%)/6 (60%)
HIV viral load	—	undetectable	undetectable
HAART scheme		23/23	10/10
NRTI + NNRTI	—	12/23 (52%)	4/10 (40%)
NRTI + PI	—	9/23 (39%)	6/10 (60%)
NRTI + NNRTI + PI	—	1/23 (4.3%)	0
NRTI + INI	—	1/23 (4.3%)	0
Dengue Classification, WHO, 2009			
DwoWS	6/20 (30%)	14/23 (60%)	—
DwWS	9/20 (45%)	8/23 (34%)	—
SD	5/20 (25%)	1/23 (4,3%)	—
IgM anti-DENV positive	19/20 (95%)	19/23 (82%)	—
NS1anti-DENV positive	15/20 (75%)	16/23 (69%)	—
Previous dengue infection(IgG positive)	16/20 (80%)	15/23 (65%)	—
DENV Serotype	10/20 (50%)	7/23 (30%)	—
	DENV-1:7	DENV-1:4	—
	DENV-2:1	DENV-2:0	—
	DENV-3:0	DENV-3:0	—
	DENV-4:2	DENV-4:3	—
Leukocytes/mm^3^	4275 (3300–4900)	3950 (2940–5130) ^+^	6850 (3950–8400)
Lymphocytes/mm^3^	2245 (1154–3124)	1842 (823–2525)	—
Monocytes/mm^3^	357.5 (258–552)	265 (171–1017)	—
CD4 absolute counts	267.0 (307.8–715.4)	203.4 (192.8–473.3)^+^	408.9 (293.7–1265)
CD4%	26.9 (22.9–39.0)^≠^	20.3 (14.3–26.4)^+^	35.4 (21.0–41.0)
CD8 absolute counts	312.6 (205.7–725.7)	256.1 (203.3–1137)	401.5 (345.3–1021)
CD8%	21.6 (14.4–30.8)^≠^	35.1 (29.5–51.9)^+^	26.8 (19.1–43.0)
CD4/CD8 ratio	1.6 (0.8–2.1)^≠^	0.5 (0.3–0.7)	1.3 (0.5–2.0)
Hematocrit %	39.9 (38.0–41.0)	39.5 (37–41.0)	40.2 (33.7–44.9)
Platelets counts/mm^3^	114.5 (38.0–168.0)^∝^	158.0 (131.0–185.0)	215.5 (137.0–285.0)
AST IU/L	47.7 (95.8–115.0)	59.5 (33–106.0)	—
ALT IU/L	85.5 (57.0–122.0)^≠^	60.0 (46.0–80.0)	—
Albumin g/dL	3.0 (3.0–4.0)	3.0 (3.0–3.0)	—

Data are median value (interquartile range) or no. (%) of patients.

ALT: alanine aminotransferase; AST: aspartate aminotransferase.

Kruskal Wallis Test with Dunn’s multiple comparisons test.

^≠^DENV vs. DENV/HIV *p < 0.05.

^+^DENV/HIV vs. HIV *p < 0.05.

^∝^DENV vs. HIV **p < 0.01.

DwoWS: Dengue without warning signs;

DwWS: Dengue with warning signs;

SD: Severe Dengue.

NRTI-nucleoside reverse transcriptase inhibitors.

PI-protease inhibitors.

NNRTI- non-nucleoside reverse transcriptase inhibitors.

INI-integrase inhibitors.

### Characterization of CD4 and CD8 T lymphocytes subsets from DENV and DENV/HIV coinfected patients

**Figure 1:**
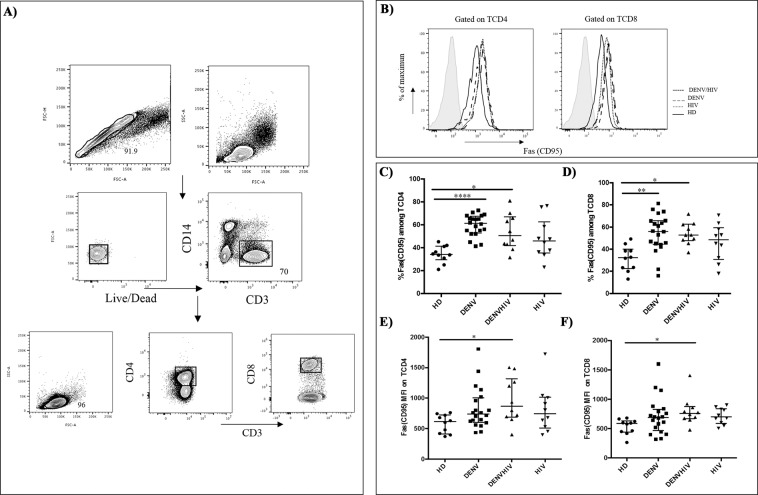
Increased frequencies of Fas/CD95 death receptor in T lymphocytes of patients infected with DENV, DENVHIV and HIV. PBMCs from healthy subjects, DENV, DENVHIV and/or HIV infected patients were isolated and labeled as described in Materials and Methods section. (**A**) Gating strategies: Exclusion of doublets, dead cells and CD14+ monocytes were applied and then lymphocytes were gated according on FCS and SSC. T lymphocytes CD3^+^CD4^+^ and/or CD3^+^CD8^+^ were selected in the analysis. **(B)** Representative histograms showing the *ex vivo* Fas/CD95 levels in CD4 and CD8 T cell subsets from one DENV, DENVHIV, HIV and a healthy control donor. Isotype controls were used in all analysis (Gray histogram). Scatter plots comparing frequencies of CD95-expression among CD4 T (**C)** and CD8 T (**D**) cells subsets. Scatter plots comparing Fas/CD95 MFI on CD4 T (**E**). Scatter plots comparing Fas/CD95 MFI on CD4 T **(F)**. Horizontal bars indicate the median values, interquartile range for each group population (HD = 10, DENV = 20, DENVHIV = 10 and HIV = 10). Values (medians with interquartile range) were submitted to Kruskal Wallis test with Dunn’s multiple comparisons test (*p< 0.05, **p < 0.01 and ****p< 0.0001).

The frequencies of CD4 and CD8 T-cell subsets were analyzed by flow cytometric using specific monoclonal antibodies. Figure 1A shows gating strategy for analysis of T -cell subsets. The median of CD4 T absolute counts is lower in DENV/HIV coinfected patients as compared to HIV (p < 0.05). Additionally, DENV/HIV coinfected patients had a higher CD8 T percentages and low CD4/CD8 ratio compared to DENV group (p < 0.05) (Table 1).

### DENV and DENV/HIV coinfected patients present a higher frequency of T lymphocytes expressing Fas/CD95 death receptor

We have already shown that DENV infection induces up regulation of death receptor Fas/CD95 in both CD4 T and CD8 T- cell subsets^13^. In order to evaluate whether Fas/CD95 cell surface receptor plays a role during coinfection, we compared Fas/CD95 expression in T-cell subsets from different groups (Fig. 1B). The frequencies of Fas/CD95 gated on CD4 T lymphocyte were prominently higher in DENV and DENV/HIV coinfected patients compared to HD [HD: 34.15 (25–42), n = 10; DENV 61.2 (55–65.3), n = 20; DENV/HIV 50.6 (38.6–70.2), n = 10; HIV 45.8 (33.7–75.4), n = 10] (Fig. 1C). Similarly, Fas/CD95 expression among CD8 T lymphocytes was higher in DENV and DENV/HIV coinfected patients compared to HD [HD: 32.2 (20–44), n = 10; DENV 56 (45–65), n = 20; DENV/HIV 52.7 (45.2–63), n = 10; HIV 48 (26.2–63.5), n = 10] (Fig. 1D). In addition, the median fluorescence intensity (MFI) of Fas/CD95 on CD4 and CD8 T-cell subsets in coinfected patients was significantly higher than in HD (Fig. 1E,F). CD4 T cells expressing Fas/CD95 are inversely associated with CD4 T cell count in DENV monoinfection (r = 0.5, p = 0.0403).

### *Ex vivo* Bcl-2 levels are greatly reduced in DENV monoinfection and DENV/HIV coinfection

**Figure 2:**
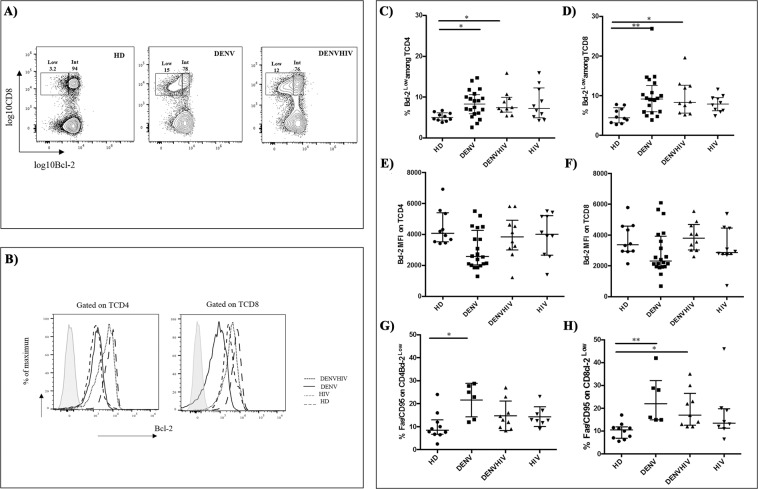
Down regulation of Bcl-2 expression in T lymphocytes from DENV and DENVHIV coinfected patients. **(A)** Representative contour plots showing *ex vivo* Bcl-2 levels in CD8 T cells from DENV, DENVHIV, HIV and a healthy control donor (HD). Cells were gated as in Fig. 1. Selected Bcl-2^low^ and Bcl-2^int^ regions are shown. **(B)** Representative histograms showing the *ex vivo* Bcl-2 levels in CD4 and CD8 T cell subsets from DENV, DENVHIV, HIV and HD. Isotype controls were used in all analysis (Gray histogram). Scatter plots comparing percentages of Bcl-2^Low^ among CD4 T (**C)** and CD8 T-cell subsets (**D)** from DENV, DENVHIV, HIV and HD. Scatter plots comparing Fas/CD95 MFI on CD4 T **(E)**. Scatter plots comparing Fas/CD95 MFI on CD8 T (**F)**. Frequency of Fas/CD95 expressed among CD4 T (**G)** or CD8 T (**H)** cells subsets expressing low levels of anti-apoptotic protein Bcl-2 (Bcl-2^Low^) between HD and groups of study. Horizontal bars indicate the median values, interquartile range for each group population (HD = 10, DENV = 20, DENVHIV = 10 and HIV = 10). Values were submitted to Kruskal Wallis test with Dunn’s multiple comparisons test (* p < 0.05, **p <  0.01).

Studies from HIV infected patients demonstrated down-regulation of the anti-apoptotic protein Bcl-2 (Bcl-2^low^) on CD8 T cells making then susceptible to apoptosis *in vitro*. Most CD8 T cells from non-HIV infected individuals expressed homogeneous (intermediary or normal) levels of Bcl-2^20^. Besides, activated CD4 T and CD8 T cells expressed low levels of Bcl-2 during dengue infection as demonstrated previously by our group^7,13^. In this way, this prompted us toinvestigate whether coinfection could influence the levels of Bcl-2 protein expression in T- cell subsets. Figure 2A demonstraterepresentative contour plots of Bcl-2 protein expression on CD8 T –cell subset from a representative HD, DENV and DENV/HIV. In addition, representative histograms of *ex vivo* Bcl-2 protein expression in CD4 T and CD8 T lymphocytes from HD, DENV, DENV/HIV and HIV are also shown (Fig. 2B). CD4 T lymphocytes expressed significantly lower levels of intracellular Bcl-2 protein (Bcl2 ^low^) during Dengue monoinfection and DENV/HIV coinfection as compared to HD [HD: 4.9 (4.1–6.4), n = 10; DENV 8.2 (6.0–10), n = 20 and DENV/HIV 7.3 (5.3–10.7), n = 10] (Fig. 2C). The intermediary/normal levels of Bcl-2 (Bcl-2^Int^) frequency among CD4 T were not statistically different between groups: [HD: 87 (81.4-89.7), n = 10; DENV 87.6 (77–90), n = 20; DENV/HIV 86 (77–90), n = 10; HIV 90.2 (83–93), n = 10]. Similarly, CD8 T lymphocytes expressed significantly lower levels of intracellular Bcl-2 protein (Bcl2 ^low^) during dengue infection and coinfection with HIV [HD: 4.4 (2.9–7.6), n = 10; DENV 9.2 (6.0–10.8) n = 20; DENV/HIV 8.3 (5.5–13), n = 10; HIV 7.9 (5.9–10), n = 10] (Fig. 2D). Intermediary/normal levels of Bcl-2 (Bcl-2^Int^) frequency among CD8 T lymphocytes were not statistically different between groups: [HD: 88 (86–93), n = 10; DENV 85 (83–91), n = 20; DENV/HIV 85.4 (73–89), n = 10; HIV 87.8 (84–89), n = 10]. No significant difference in the MFI of Bcl-2 protein on CD4 T and CD8 T cells among study groups were found, although we observed a tendency of decreased Bcl-2 levels in DENV infected patients as compared to HD: CD4 T [HD: 4067 (3453–5550), n = 10; DENV 2573 (2100–3690), n = 20, p = 0.0486] (Fig. 2E); CD8 T [HD: 3381 (2930–4617), n = 10; DENV 2310 (1960–3600), n = 20, p = 0.0480] (Fig. 2F).

### Down-regulation of Bcl-2 protein concomitant with the increased Fas/CD95 expression in T lymphocytes in Dengue infection and coinfection with HIV

In order to evaluate a possible association of extrinsic and intrinsic apoptotic pathways, we analyzed the double expression of  death receptor Fas/CD95 and the anti-apoptotic molecule Bcl-2 on T-cells in a smaller number of patients. As shown in Fig. 2G, Fas/CD95 was up regulated predominantly on the surface of CD4 T cells expressing low levels of Bcl-2 in DENV infected patients compared to HD: [HD: 8.4 (6.5–16), n = 10; DENV 23.9 (12–28.7) n = 6; DENV/HIV 13 (9–23), n = 9; HIV 12.8 (9–23.1), n = 8] (Fig. 2G). Similarly, the percentage of Fas/CD95 on CD8 T Bcl-2^Low^ was higher in DENV and DENV/HIV as compared to HD: [HD: 10.5 (6.0–13), n = 10; DENV 22 (14–42) n = 6; DENVHIV 17 (12–30), n = 9; HIV 13.5 (6.4–46), n = 8] (Fig. 2H). Besides, Fas/CD95 expression was analyzed on CD4 T and CD8 T -cell subsets expressing normal levels of Bcl-2 (Bcl-2^Int^) and the medians were not significant different between groups analyzed: CD4 T Bcl-2^Int^ [HD: 16.5 (12–25), n = 10; DENV 21.7 (17–35) n = 6; DENV/HIV 17.3 (8.9–36), n = 9; HIV 22.5 (6.8–42), n = 8]; CD8 T Bcl-2^Int^ [HD: 11.9 (8.1–19), n = 10; DENV 16 (6.5–25) n = 6; DENV/HIV 14 (8.0–19), n = 9; HIV 14.5 (4.6–38), n = 8].

### Characterization of monocyte subsets during DENV/HIV coinfection

**Figure 3:**
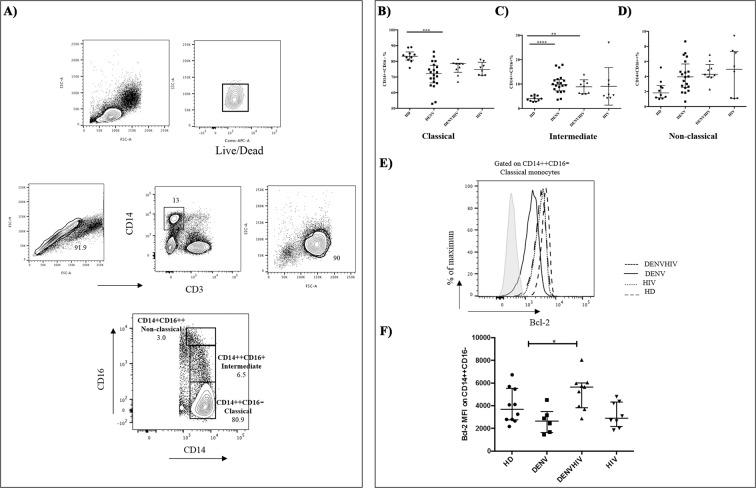
Intermediate CD14^+^CD16^+^ monocytes are expanded during DENV and DENVHIV co-infection. PBMCs from healthy subjects, DENV, DENVHIV and/or HIV patients were isolated and labeled as described in Materials and Methods section. **(A)** Gating strategies: Exclusion of doublets, dead cells and CD3^+^ lymphocytes were applied and then monocytes were gated according on FCS and SSC. The gating strategy for analysis of classical CD14^++^CD16^−^, intermediate CD14^++^CD16^+^ and non-classical CD14^+^CD16^++^ monocyte subsets of representative DENV infected patient is indicated in the left panel. Scatter plots comparing frequencies of classical CD14^++^CD16^−^ (**B)**, intermediate CD14^++^CD16^+^ (**C)** and non-classical CD14^+^CD16^++^ (**D)** monocyte subsets. (**E)** Representative histograms showing the *ex vivo* Bcl-2 MFI in classical CD14^++^CD16^−^ from DENV, DENVHIV, HIV and HD. Isotype controls were used in all analysis (gray histogram). **(F)** Scatter plots comparing Bcl-2 MFI on classical monocytes CD14^++^CD16^−^. Horizontal bars indicate the median values, interquartile range for each group population (HD = 10, DENV = 20, DENVHIV = 10 and HIV = 10). Values were submitted to Kruskal Wallis test with Dunn’s multiple comparisons test (*p  < 0.05, ** p < 0.01, ***p < 0.001 and ****p<0.0001).

According to CD14 and CD16 expression, 3 distinct subpopulations are characterized: classical (CD14^++^CD16^−^), intermediate (CD14^++^CD16^+^) and non-classical (CD14^+^CD16^++^) subsets^21^ and we characterized them phenotypically using gating strategy, as shown in Fig. 3A. As monocytes are primary targets for DENV infection^22^, we analyzed monocyte phenotypes during DENV or DENV/HIV coinfected individuals. As seen in Fig. 3B,C CD14^++^CD16^−^ classical monocytes were decreased during DENV infection as compared to HD: [HD: 83 (80–88), n = 10; DENV 72 (67–76.3) n = 20; DENV/HIV 78.5 (71–79), n = 9; HIV 74.7 (70–81), n = 8]. Furthermore, DENV infection as well as coinfection with HIV resulted in significant increase of CD14^++^CD16^+^ intermediate monocytes [HD: 3.8 (2.8–5.3), n = 10; DENV 9.7 (7.9–11.5) n = 20; DENV/HIV 9.3 (6.1–11.4), n = 9; HIV 5.5 (4.2–27), n = 8]. No statiscally significant difference was found in CD14^+^CD16^++^ non-classical monocytes frequencies among study groups: [HD 1.8 (1.07–3.3), n = 10; DENV 3.9 (2.6–4.6) n = 20; DENV/HIV 4.3 (3.9–6.1), n = 9; HIV 4.9 (1.01–9.4), n = 8] (Fig. 3D).

### Death receptor Fas/CD95 and anti-apoptotic molecule Bcl-2 expressions on monocytes subsets in DENV and DENV/HIV infected individuals

To characterize Fas/CD95 and Bcl-2 expressions in the monocytes subsets, we analyzed the MFI of death receptor Fas/CD95 and anti-apoptotic molecule Bcl-2. As observed in Fig. 3E-F, MFI of Bcl-2 on CD14^++^CD16^−^ classical monocytes in DENV monoinfected patients was significantly lower as compared to DENV/HIV coinfected ones: [HD: 3667 (2657–5688), n = 10; DENV 2658 (1459–4505) n = 6; DENV/HIV 5646 (3681–6048), n = 9; HIV 2901 (1850–4800), n = 8]. Lower Bcl-2 expression on CD14^++^CD16^+^ intermediate monocytes was observed in DENV infected patients, but it did not reach statistical significance between groups: [HD: 4812 (2266–7723), n = 10; DENV 2925 (1491–7553) n = 6; DENV/HIV 6434 (4049–10935), n = 9; HIV 2772 (1489–7857), n = 8]. No statically significant difference was found in the Bcl-2 expression among CD14^+^CD16^++^ non-classical monocytes [HD: 4634 (1774–10936), n = 10; DENV 5387 (2690–11923) n = 6; DENV/HIV 9063 (3328–12420), n = 9; HIV 3932 (1496–18671), n = 8]. Additionally, 3 monocyte subsets were not altered in their Fas/CD95 expression profile (data not shown).

### Up regulation of apoptotic and anti-apoptotic proteins suggests cell death modulation of PBMCs in HIV treated patients at acute Dengue infection

The analysis of apoptosis-related proteins expression profile is essential for understanding signaling molecules roles and their involvement in programmed cell death mechanisms during disease states. To investigate changes in apoptosis-related proteins, we evaluated the relative expression levels of 35 apoptosis-related proteins simultaneously detected in a single sample. We assessed the following representative samples: heathy donor -HD n = 2; DENV mono-infected patients n = 8; DENV/HIV coinfected patients n = 3 and HIV infected controls n = 2. The results allowed us to compare those different groups and estimate wich proteins would have changed their expression. Among the 35 apoptosis -related proteins analyses, 13 (37%) reached very lower density values and were not evaluated. However, the other 22 (62.8%) proteins had their expression increased or decreased.

**Figure 4:**
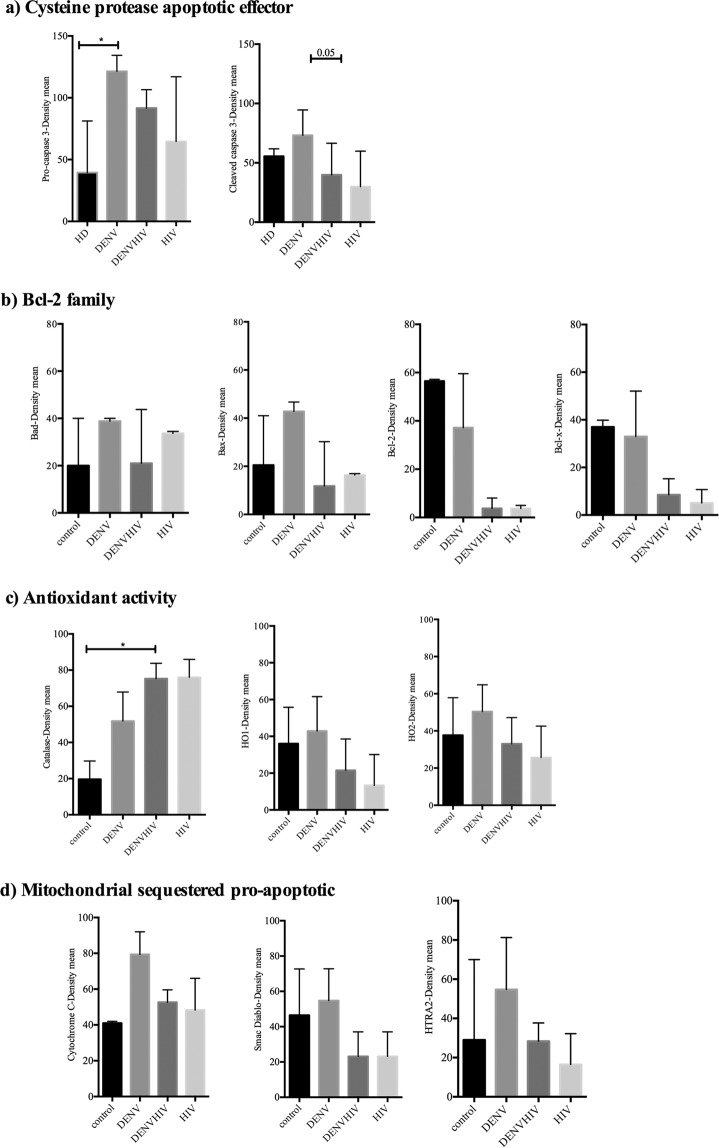
Expression of apoptotic proteins in PBMC lysates of HD, DENV, DENVHIV and HIV. (**A**) Cysteine protease apoptotic effector: Pro caspase 3 and Cleaved caspase 3. (**B**) Bcl-2 family: Bad, Bax, Bcl-2 and Bcl-x (**C**) Antioxidant molecules: Catalase, HO1 and HO2. (**D**) Mitochondrial sequestered pro-apoptotic: Cytochrome C, Smac /DIABLO and HtRA2/Omi. Values were submitted to Kruskal Wallis statistical test (*p < 0.05). PBMCs were isolated and purified by density gradient centrifugation (Ficoll-Paque Premium, GE Healthcare Biosciences). Seventeen of 35 cellular apoptotic proteins expression profiles were analyzed on cell lysed obtained by protease inhibitor cocktail and lysis buffer treatments. Afterwards, Human Apoptosis Array Kit was applied. The lysate volumes were adjusted, according to the manufacturer. Proteins expression was detected as spots in nitrocellulose membranes. Data of each group of apoptotic proteins stratified according to their function/family and cell location were presented as columm graphs.

Our study failed to demonstrated statistically significant differences in most proteins analyzed probable due small sample size. However, data analysis of pro-caspase 3 demonstrated an increased expression in DENV monoinfected compared with HD [HD: 39 (10–69) n = 2; DENV 125.9 (101–136) n = 8; median-minimum/maximum; Kruskal-Wallis test with Dunn’s multiple comparisons test p > 0,05] (Fig. 4A). Caspases are divided into two groups, the initiators and the effectors. They are expressed in inactive form (pro-caspases) and, when activated, (cleaved caspase) are involved in early and late regulatory apoptosis events. Effector caspase 3 is proteolytically activated in a cascade leading to cell disintegration^3^. No statistically significant differences were found in cleaved caspase 3 among groups, although the trend is toward increased medians in DENV monoinfected compared to coinfected [DENV 72.6 (39–105) n = 8; DENV/HIV: 51.2 (9.5–58.9) n = 3; median-minimum/maximum; Kruskal-Wallis test with Dunn’s multiple comparisons test p = 0,05].

The oxidative stress production caused by the reactive oxygen species (ROS) accumulation is another manner of apoptosis induction^23^. Excessive accumulation of intracellular hydrogen peroxide (H_2_O_2_) generates oxidative stress and apoptosis trigger. The antioxidant catalase enzyme captures H_2_O_2_ and converts it into water and oxygen stimulating cell survival^24^. The anti-oxidant molecule catalase, was upregulated in DENV, DENVHIV as well as HIV infected. DENV/HIV coinfected patients had significantly higher expression of catalase compared to HD [DENVHIV 72 (69-84.9) n = 3 ; HD: 19.5 (12.5–26.8) n = 2; median-minimum/maximum; Kruskal-Wallis test with Dunn’s multiple comparisons test p > 0,05]. (Fig. 4C).

**Figure 5:**
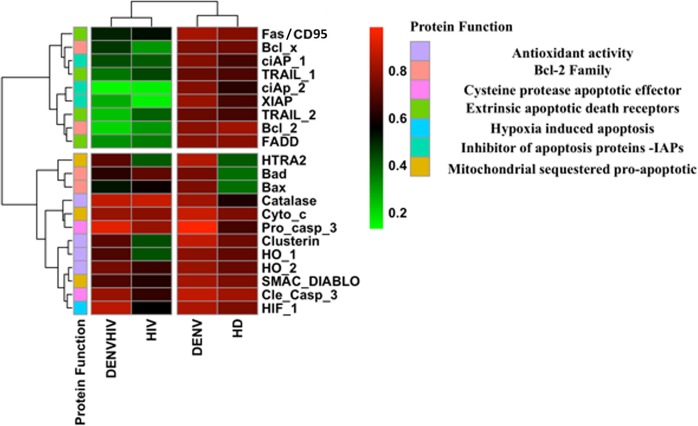
Heat map data cluster analysis of apoptotic protein expresion in treated HIV patients during acute dengue infection. Heatmaps are shown for log10-fold change of individual protein expression. The analysis approach combined clustering methods, which group samples together based on the similarity of their protein expression pattern to identify or compare possible biological signatures associated with study groups. Data were shown in a grid where each line represents a protein and each column represents a group of HD, DENV, HIV or DENVHIV samples. The color and intensity of each box were applied to represent changes of protein expression: Red represents up-regulated protein levels and green represents down-regulated ones. For each protein, different color boxes were also created to distinguish effector functions related to apoptotic pathways. Hierarchal clustering was performed for all samples using R. Healthy donors (HD n = 2), Dengue infected patients (DENV, n = 8), DENV/HIV coinfected (DENV/HIV, n = 3) and HIV treated controls (HIV, n = 2).

As previously mentioned, no significant differences in the level of most apoptotic proteins were found between studied groups, probable due small sample size. However, we built a two-dimensional heat map with hierarchical clustering that grouped the expression of the apoptotic-cell markers in Y dimension and segregated the HIV, DENV, DENV/HIV and HD groups in the X dimension (Fig. 5). Additionally, to confirm the grouping and to determine the cluster robustness observed in Fig. 5, we ran a bootstrapped clustering (n = 1000 replicates). Here, we confirmed that separated clusters in the image had support ( p < 0.05). As observed in Fig. 5, downregulation of pro-apoptotic proteins (Fas/CD95, TRAIL1, TRAIL2 and FADD), anti-apoptotic proteins (Bcl-2, Bcl-x) and the inhibitors of apoptosis proteins -IAPs (ciAP1 ciAP2, XIAP) were found in DENV/HIV coinfected and HIV infected groups. On the other hand, the pro-apoptotic proteins Fas/CD95, TRAIL1, TRAIL2 and FADD were upregulate in most DENV infected patients. Similarly, the IAPs were upregulated during dengue monoinfection. In addition, DENV and DENV/HIV were characterized as having high levels of Bad and Bax, , although only DENV/HIV coinfected and HIV infected groups showed increased Bax/Bcl-2 ratios [DENV/HIV 5.3 (1.3–37.7) n = 3; DENV 0.9 (0.86–11.4) n = 8 and HIV 4.7 (3.4–6.0) n = 2 medium- Minimum -maximum]. Confirming the pattern previously observed in Fig. 4c, catalase antioxidant protein was upregulated in DENV, DENV/HIV and HIV.

A principal component analysis (PCA) was performed to evaluate the grouping of each individual from the studied groups in a multidimensional space. The PCA1 explained 69.77% of the variation. The variables with more contribution for the separation along PCA1 were ciAp-2, Bcl-2, XIAP, Bcl-x, FADD, ciAP-1, and TRAIL-2. Along the PCA2 the analytes that contributed more were Bad, Bax, and Pro caspase 3 (see Supplementary Table S1 and Supplementary Fig. S1). Along the PCA1 dimension we observed a separation of all individuals from DENV/HIV and HIV groups. One of the HD presented low levels of Bad, Bax and pro caspase 3 (seen as the point opposed to the Bad, Bax and pro caspase 3 arrows along X axis). Two individuals of group monoinfected by DENV presented similar responses to DENV/HIV and HIV groups.

## Discussion

Dengue and AIDS are considered public health emergency with higher incidence of morbidity and mortality. Brazil has been plagued by a triple epidemic caused by arboviruses (Dengue, Zika and Chikungunya) of worldwide impact^25^. In this scenario, DENV and HIV coinfection is a reality and it is unclear whether HIV infection could increase DENV severity. Literature data indicate that most coinfected patients had mild outcomes of dengue. Corroborating, the present data demonstrated that most coinfected patients had mild symptons confirming our previous data^14^. More recently, Hotzz *et.al*., reported that DENV infection in ART- treated  HIV patients was associated with reduced vascular instability and liver damage^26^. However, coinfected patients could have severe dengue outcomes^27^.

In this study, quantitative and qualitative changes in T cells and monocytes subsets during DENV infection in HIV treated patients were evaluated. We found significantly decrease in CD4 and CD8 T- cell subsets confirming earlier results with infected Brazilian patients^28^. Interestingly, decreased CD4/CD8 T ratio and increased in the CD8 T percentages were observed in coinfected patients as compared to DENV infected. The ART era enable immune restoration and improved AIDS related and non-AIDS related morbidity as well as mortality turning HIV/AIDS into chronic disease. Despite ART effectiveness, late ART initiation is a Brazilian challenge^29–31^. A significant number of individuals start HIV/AIDS care at later stage of infection (later presenters) and usually present low T CD4 counts even with complete viral suppression. Several factors are associated with T CD4 recovery including age, specific ART regimens, comorbidities and coinfections^32^. In fact, CD4/CD8 ratio has been considered as a biomarker for treated HIV patients^33^. It is not clear whether altered CD4/CD8 T ratios could lead to dengue severity in ART treated HIV patients. However, besides decreased CD4/CD8 ratios, DENV/HIV coinfected patients evolved with a good prognosis.

Studies demonstrated the occurrence of massive apoptosis in lymphocytes during the acute phase of DENV infection^34,35^. In agreement with this, we found that frequencies of CD4 and CD8 T cells expressing Fas/CD95 were increased during DENV and DENV/HIV coinfection. Furthermore, Bcl-2 levels on T cells were greatly reduced in DENV and DENV/HIV coinfected compared to healthy individuals. More importantly, the Fas/CD95 upregulation on both CD4 and CD8 T cells expressing low levels of Bcl-2 were increased during DENV infection and also in CD8 T cells of co-infected patients suggesting apoptotic events on T lymphocytes. In fact, we found an inverse correlation between CD4 T absolute counts and CD4 T cells expressing Fas/CD95 in DENV monoinfection. During the extrinsic apoptotic pathway, the interaction between death receptor Fas/CD95 agonist and its natural ligand FasL promotes the recruitment of adapter proteins that in turn interact with caspase 8 to trigger the initial step of apoptosis^36^. We previously described phosphatidylserine exposure and DNA fragmentation on T lymphocytes besides increased Fas/CD95 death receptors expression and decreased Bcl-2 expression in naturally DENV infected patients^13^. Moreover, DENV-specific CD8 T cells were susceptible to apoptosis as demonstrated by Bcl-2 downregulation, Fas/CD95 upregulation and DNA fragmentation^37^. CD4 T cell depletion is more pronounced in coinfected patients suggesting that an apoptotic extrinsic pathway may be contributing to the T lymphocyte depletion. Persistent coinfection between GB virus C (GBV-C) and HIV lead to a slower disease progression and mortality in HIV infected patients^38^. Fas/CD95 expression was lower in GBV-C and non-treated HIV coinfected patients suggesting that reduced Fas/CD95-mediated apoptosis of T cells might be responsible for beneficial effect of GBV-C coinfection. Moenkemeyer and colleagues suggested that GBV-C inhibit host cell apoptosis and consequently maintain chronic GBV-C infection. In agreement with our study, Fas/CD95 expression on T cells were not significantly different in patients receiving ART and degree of Fas/CD95-expressing lymphocytes was comparable between GBV-C co-infected and GBV-C infected^39^. A recent study demonstrated that apoptosis of platelets were similar between DENV infected and coinfected with HIV as evidenced by phosphatidylserine exposure, mitochondrial membrane potential and caspase -9 activation^26^.

Blood monocytes have been divided into 3 subsets based on relative expression of CD14 and FCγIII receptor CD16 on classical monocytes (CD14^++^CD16^−^), intermediate (CD14^++^CD16^+^) and non-classical monocytes (CD14^+^CD16^++^). CD14^++^CD16^−^ classical monocytes presented high phagocytic activity and they are critical for initial inflammatory responses while CD14^++^CD16^+^ intermediate monocytes are associated with inflammatory response, and CD14^+^CD16^++^ non-classical are considered as patrolling monocytes^21^. Monocyte activation has been reported during DENV and HIV infections and they are associated with disease pathogenesis^22,40^. Our results demonstrated significant changes of monocyte subsets during DENV and DENV/HIV coinfection. As demonstrated previously^41,42^, we found that in the acute DENV infection, CD14^++^CD16^−^ classical monocytes were significantly decreased while intermediate were increased. Similarly, an expansion of intermediate monocytes and a reduction of classical monocytes during coinfection were found. Monocytes play important roles in the innate and adaptive response and are key players in mediating anti-DENV immune responses. They are the main target cells of DENV infection and after activation produce pro-inflammatory mediators involved in dengue severity^11^. Perturbations of monocyte subsets were also found in treated HIV patients^43,44^. In agreement we observed that some HIV treated individuals presented increased percentages of intermediate and non-classical monocytes suggesting chronic immune monocyte activation despite virus suppression after ART. More importantly, we report, for the first time (to our knowledge) that DENV/HIV coinfected patients showed increased frequencies of CD14^++^CD16^+^ intermediate monocytes. Interestingly we found that, in the context of DENV infection, the MFI of Bcl-2 on CD14^++^CD16^−^ classical monocytes were significantly lower as compared to DENV/HIV coinfection. Activation of apoptosis pathway by DENV infected target cells have been described previously^11^. Our group and others have previously shown that DENV is capable of apoptosis induction in infected monocytes by extrinsic and intrinsic pathways^12,45^. Is unknown whether apoptosis could occur as directed mechanism of viral dissemination and evasion or simply represents an appropriate host response to limit virus replication. More recently, it was demonstrated that a pro- survival protein, Bcl-xl, is involved in the survival of DENV, Zika virus (ZIKV) and Japanese encephalitis virus (JEV) HuH7 infected cells. Suzuki and colleagues proposed that Flavivirus infection induces delayed cell apoptosis and consequently virus spread to neighboring cells leading to higher viral loads and disease severity. Bcl-xL inhibition accelerates apoptosis leading to enhanced viral clearance through macrophage phagocytosis^46^.

Screening of 35 apoptosis-related proteins expression on PBMCs of study groups showed upregulation of pro caspase 3 and cleaved caspase 3 in DENV monoinfected group. Additionally, pro-apoptotic proteins involved in extrinsic apoptosis (Fas/CD95, TRAIL1, TRAIL2 and FADD) were down regulated in DENV/HIV and HIV, whereas these molecules were not downregulated in DENV infected. Our study confirmed extrinsic apoptotic death receptor Fas/CD95 and Bcl-2 family involvement in apoptosis regulation during DENV infection. However, other regulatory molecules might be involved in apoptosis of activated cells during infection. TRAIL appears to be capable of apoptosis inducement on T lymphocytes in DENV infected patients^35^. Matsuda *et al*. (2005) show that hepatocytes express TRAIL-RII and produce soluble TRAIL during DENV infection, undergoing apoptosis^47^. Endothelial cells infected by DENV-2 showed decreased TRAIL expression suggesting  evasion of TRAIL induced apoptosis^48^. Taken together, these results indicate that differential expression of apoptotic proteins observed in dengue monoinfection may be involved in apoptosis susceptibility during infection as reported previously^11^.

Our results indicate that pro-apoptotic proteins (Fas/CD95, TRAIL1, TRAIL2 and FAD), anti-apoptotic Bcl-2, Bcl-x) and IAPs (ciAP1 ciAP2, XIAP) were downregulated in DENV/HIV and HIV. It has been reported that HIV encoded proteins induce pro- and anti-apoptotic activities^49,50^. On the other hand, ART reduce direct cytopathic effect of viral proteins, immune responses to viral antigens and decreases spontaneous T cell apoptosis in successfully treated individuals^51^. PI as well as NRTI regimens induce intrinsic anti-apoptotic activity and consequently reduce lymphocyte apoptosis^52,53^. Indeed, most HIV patients of our study received PI and NRTI therapies. Although a reduction in immune activation and apoptosis can be observed after initiation of ART therapy^54^, viral latent reservoirs could support immune cell activation and CD4 T cell apoptosis^55^. In this context, we observed that Bad and Bax were upregulated not only in DENV monoinfection but also in DENV/HIV coinfection.

Smac/DIABLO and michocondrial serine protease HtrA2/Omi are reported to promote apoptosis by inhibiting IAP activity^56,57^. The IAP family of proteins  are important regulators of both intrinsic and extrinsic apoptosis pathways^58^. The XIAP and survivin remain the better-known members of this family^59^. Our data also suggested an opposite balance between an increase of those apoptotic proteins and downregulation of IAPs proteins expressions during coinfection.

Of particular interest were catalase antioxidant protein, found to be significantly upregulated in DENV/HIV coinfection. Reactive oxygen species (ROS) accumulation generate oxidative stress and induce apoptosis by increasing the collapse of mitochondrial membrane electric potential, an important step in early apoptosis. Also, ROS react with many biological molecules, including lipids, proteins and carbohydrates, causing loss of cellular integrity and cellular dysfunctions as well. We already show that catalase, an antioxidant protein, is up-regulated in severe dengue patients, indicating a possible scenario of oxidative stress in response to infection^13^. Here, for the first time, we observed data that suggests a similar scenario during Dengue/HIV coinfection.

There may be some possible limitations in this study. Quantification of DENV viral load in the serum of  coinfected patients was not possible in our study. Although, we observed that levels of circulating NS1 protein were not different between DENV monoinfected and DENV/HIV coinfected (data not shown). In fact, Hottz and colleagues demonstrated no difference in the DENV viremia between DENV infection or coinfection with HIV^26^. Unfortunately, due limited sample size we could not evaluate spontaneous apoptosis *in vitro*.

Our findings have to be interpreted carefully specially concerning to limited sample size. However, we demonstrated here several evidences supporting the concomitant up regulation of apoptosis in DENV/HIV coinfected patients corroborating previous data observed during DENV infection^13^. Importantly, pathogenesis of ART treated HIV patients with acutedengue disease remains largely unknown. In the present study, we report, for the first time that DENV/HIV coinfected patients show increased frequencies of apoptotic molecules on PBMCs. Further *in vivo* and *in vitro* studies are needed to evaluate the outcomes of coinfection in risk areas of Brazil, especially due co-circulation of other arboviruses of medical importance such as ZIKV and chikungunya virus (CHIKV). Further understanding about regulation of extrinsic and intrinsic apoptotic pathways during HIV coinfection is necessary to develop new therapies for both infections.

## Supplementary information

Supplementary information 1. 

Supplementary information 2
