# Supplementary material for: Apoptosis characterization in mononuclear blood leukocytes of HIV patients during dengue acute disease
Source: Sci Rep. 2020 Apr 14;10:6351. doi: 10.1038/s41598-020-62776-4 (PMC7156518; doi:10.1038/s41598-020-62776-4)
Supplement: Supplementary file 1 — Supplementary information 1. [file 41598_2020_62776_MOESM1_ESM.docx]

Apoptosis characterization in mononuclear blood leukocytes of HIV patients during dengue acute disease**.**

Amanda Torrentes-Carvalho

Tamiris Azamor

Luciana Santos Barbosa

Eugênio Damacedo Hottz

Mariana Gandini

Juan Camilo Sánchez-Arcila

Fernando Augusto Bozza

Rivaldo Venâncio da Cunha

Luzia Maria de Oliveira Pinto

Paulo Vieira Damasco

Elzinandes Leal de Azeredo

Table 1. Principal Component Analysis (PCA). Loading values (contribution for separation in the multivariate space) for PCA1 and PCA2.

| **Analyte** | **PC1** | **PC2** |
| --- | --- | --- |
| ***ciAp-2*** | -0.9035 | 0.0727 |
| ***Bcl-2*** | -0.8113 | 0.1091 |
| ***XIAP*** | -0.7475 | 0.0444 |
| ***Bcl-x*** | -0.6807 | -0.0259 |
| ***FADD*** | -0.6534 | 0.1191 |
| ***ciAP-1*** | -0.6214 | -0.0017 |
| ***TRAIL-2*** | -0.6134 | 0.1178 |
| ***Clusterin*** | -0.5228 | -0.0985 |
| ***TRAIL-1*** | -0.5184 | 0.0748 |
| ***FAS*** | -0.5174 | 0.0233 |
| ***HO-1*** | -0.4560 | -0.0471 |
| ***HTRA2*** | -0.4384 | -0.0293 |
| ***SMAC-DIABLO*** | -0.3358 | 0.0064 |
| ***Cle Casp 3*** | -0.3112 | -0.0934 |
| ***HIF-1*** | -0.3098 | -0.1954 |
| ***Cytochrome C*** | -0.2855 | -0.1085 |
| ***Bax*** | -0.2295 | -0.5152 |
| ***HO-2*** | -0.1999 | 0.0001 |
| ***Pro casp 3*** | -0.0800 | -0.3599 |
| ***Bad*** | -0.0636 | -0.5263 |
| ***Catalase*** | 0.1611 | -0.0943 |
