# Supplementary material for: Apoptosis characterization in mononuclear blood leukocytes of HIV patients during dengue acute disease
Source: Sci Rep. 2020 Apr 14;10:6351. doi: 10.1038/s41598-020-62776-4 (PMC7156518; doi:10.1038/s41598-020-62776-4)
Supplement: Supplementary file 2 — Supplementary information 2 [file 41598_2020_62776_MOESM2_ESM.docx]

Apoptosis characterization in mononuclear blood leukocytes of HIV patients during dengue acute disease**.**

Amanda Torrentes-Carvalho

Tamiris Azamor

Luciana Santos Barbosa

Eugênio Damacedo Hottz

Mariana Gandini

Juan Camilo Sánchez-Arcila

Fernando Augusto Bozza

Rivaldo Venâncio da Cunha

Luzia Maria de Oliveira Pinto

Paulo Vieira Damasco

Elzinandes Leal de Azeredo

Figure 1. Principal Component Analysis (PCA) of apoptotic cell markers.

**
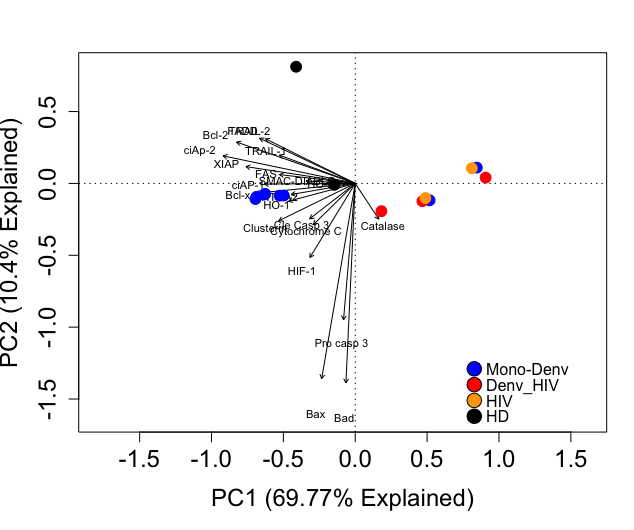
**

**Principal Component Analysis (PCA) of apoptotic cell markers**. Principal Component Analysis (PCA) was constructed in order to verify the distribution pattern of the studied analytes among the individuals. Colored points represent each analyzed patient and the variables are drawn as arrows. The length of each arrow represents the contribution to the spatial separation of the individuals. The PCA analysis were done using *vegan* using R statistical environment.
